# The Impact of Angiotensin Receptor–Neprilysin Inhibitors on Arrhythmias in Patients with Heart Failure: A Systematic Review and Meta-analysis

**DOI:** 10.19102/icrm.2022.130905

**Published:** 2022-09-15

**Authors:** Hata Mujadzic, George S. Prousi, Rebecca Napier, Sultan Siddique, Ninad Zaman

**Affiliations:** ^1^Division of Internal Medicine, Prisma Health/University of South Carolina, Columbia, SC, USA; ^2^Division of Cardiology, Prisma Health/University of South Carolina, Columbia, SC, USA; ^3^Division of Advanced Heart Failure, Prisma Health, Columbia, SC, USA; ^4^Division of Electrophysiology, Prisma Health, Columbia, SC, USA

**Keywords:** Angiotensin receptor–neprilysin inhibitor, arrhythmia, sudden cardiac death, ventricular tachycardia

## Abstract

Angiotensin receptor–neprilysin inhibitor (ARNI) use has become increasingly popular. Current guidelines recommend using ARNI therapy for heart failure with reduced (HFrEF) and preserved ejection fraction (HFpEF). As therapies become more widely available, heart failure-associated burdens such as ventricular arrhythmias and sudden cardiac death (SCD) will become increasingly prevalent. We conducted a systematic review and meta-analysis to assess the impact of ARNI therapy on HFrEF and HFpEF pertaining to arrhythmogenesis and SCD. We performed a search of MEDLINE (PubMed), the Cochrane Library, and ClinicalTrials.gov for relevant studies. The odds ratios (ORs) of SCD, ventricular tachycardia (VT), ventricular fibrillation (VF), atrial fibrillation/flutter (AF), supraventricular tachycardia (SVT), and implantable cardioverter-defibrillator (ICD) shocks were calculated. A total of 10 studies, including 6 randomized controlled trials and 4 observational studies, were included in the analysis. A total of 18,548 patients from all studies were included, with 9,328 patients in the ARNI arm and 9,220 patients in the angiotensin-converting enzyme inhibitor (ACEI)/angiotensin II receptor blocker (ARB) arm, with a median follow-up time of 15 months. There was a significant reduction in the composite outcomes of SCD and ventricular arrhythmias in patients treated with ARNIs compared to those treated with ACEIs/ARBs (OR, 0.71; 95% confidence interval, 0.54–0.93; *P* = .01; I^2^ = 17%; *P* = .29). ARNI therapy was also associated with a significant reduction in ICD shocks. There was no significant reduction in the VT, VF, AF, or SVT incidence rate in the ARNI group compared to the ACEI/ARB group. In conclusion, the use of ARNIs confers a reduction in composite outcomes of SCD and ventricular arrhythmias among patients with heart failure. These outcomes were mainly driven by SCD reduction in patients treated with ARNIs.

## Introduction

Heart failure (HF) remains a crucial contributor to recurrent hospitalization and death among individuals aged 50–89 years, with an exponential rise in prevalence over time.^1^ The American Heart Association estimates that >6 million people in the United States have a diagnosis of HF with a projected prevalence of 8 million cases by the year 2030.^2,3^ In recent years, a definite aim to develop therapeutic options for individuals with HF has been emphasized, with growing evidence and literature suggesting that novel therapies may be beneficial.

Arrhythmias are of a significant burden to those with HF diagnoses. The pathophysiology, as it relates to arrhythmogenesis, is complex but includes multifactorial manifestations of fibrosis, neurohormonal imbalance, and variability of ion channels including under- and overexpression, in addition to electrolyte abnormalities.^4,5^

One pharmacologic therapy gaining much popularity and enthusiasm is angiotensin receptor–neprilysin inhibitors (ARNIs) due to their ability to reduce the adverse manifestations of HF diagnoses. Proposed theories regarding the effect ARNIs have on the reduction of mortality and sudden death from an arrhythmia perspective are not well understood; however, circulating natriuretic peptides reduce the harmful effects of the sympathetic and renin–angiotensin systems by decreasing myocyte death, hypertrophy, fibrosis, and inflammation, which have all been implicated in arrhythmogenesis.^6^

Regarding HF with reduced ejection fraction (HFrEF), ARNIs have demonstrated a clear survival benefit as demonstrated in the Prospective Comparison of ARNI with ACEI to Determine Impact on Global Mortality and Morbidity in HF (PARADIGM-HF) trial and is now further used in the treatment of HF with preserved ejection fraction (HFpEF) due to results from the Prospective Comparison of ARNI with ARB Global Outcomes in HF with Preserved Ejection Fraction (PARAGON-HF) trial.^7,8^

The effect of ARNIs on sudden cardiac death (SCD) has been widely reported, and while meta-analyses on outcomes such as primary total mortality and HF endpoints have been reported, little data exist regarding their effect on arrhythmia.^9–11^ Given such a positive effect on reducing morbidity and mortality in HF patients, we aimed to emphasize the additional importance of reducing the burden of arrhythmia in this patient population using ARNI therapy.

## Methods

### Data sources and search strategies

We conducted a systematic review using MEDLINE (PubMed), the Cochrane Library, and ClinicalTrials.gov from inception to January 10, 2022. We used the terms “LCZ696” or “LCZ 696” or “LCZ-696” or “entresto” or “sacubitril” or “sacubitril valsartan” or “sacubitril–valsartan” or “angiotensin receptor–neprilysin inhibitor” and “heart failure” for the search strategy. The meta-analysis was conducted and performed using the Preferred Reporting Items for Systematic Reviews and Meta-analysis guidelines.^12^

### Inclusion and exclusion criteria

We included studies that incorporated the following characteristics: (1) enrolled adult patients >18 years of age with a diagnosis of HFrEF or HFpEF, (2) compared ARNI therapy to an active control group or placebo, (3) were randomized controlled trials (RCTs) or observational cohort studies, and (4) included arrhythmia endpoints. We excluded studies with duplicate data or no data of interest from an arrhythmia perspective.

### Data extraction and quality assessment

Two reviewers independently performed data extraction and quality assessments of the included studies. The data reported include the type of study, baseline characteristics of the patients, intervention, control, randomization, follow-up duration, and sample size. The outcomes of interest included SCD, ventricular tachycardia (VT), ventricular fibrillation (VF), atrial fibrillation/flutter (AF), supraventricular tachycardia (SVT), and implantable cardioverter-defibrillator (ICD) shocks. All the studies considered appropriate for the meta-analysis had their full text analyzed by 2 reviewers. In addition, data from ClinicalTrials.gov and supplemental sections were reviewed if they included the arrhythmia endpoints of our interest.

### Risk of bias assessment

All included RCTs were graded for bias using the Cochrane Handbook for Systematic Reviews of Interventions.^13^ The observational studies were graded for bias using the Newcastle–Ottawa scale.^14^ Two reviewers assessed the risk of bias for each included study.

### Statistical analysis

Summary odds ratios (ORs) with 95% confidence intervals (CIs) were calculated using a random-effects model. The random-effects model incorporates heterogeneity between trials and usually gives wider and more conservative CIs. The 95% CIs were estimated using a binomial distribution. Heterogeneity across all studies was assessed using the chi-squared and I^2^ tests. According to published guidelines, it is accepted that an I^2^ value of 25%–49% indicates low heterogeneity, 50%–74% indicates moderate heterogeneity, and >75% indicates high heterogeneity.^15^ *P* < .10 was used as an indicator for significance regarding heterogeneity, and *P* < .05 was used to indicate significance for the arrhythmia outcomes. A subgroup analysis was performed for studies with at least moderate or significant heterogeneity. The analysis was performed using Review Manager (RevMan) version 5.3 (The Nordic Cochrane Centre, The Cochrane Collaboration, Copenhagen, Denmark).

## Results

### Baseline characteristics

**Figure 1:**
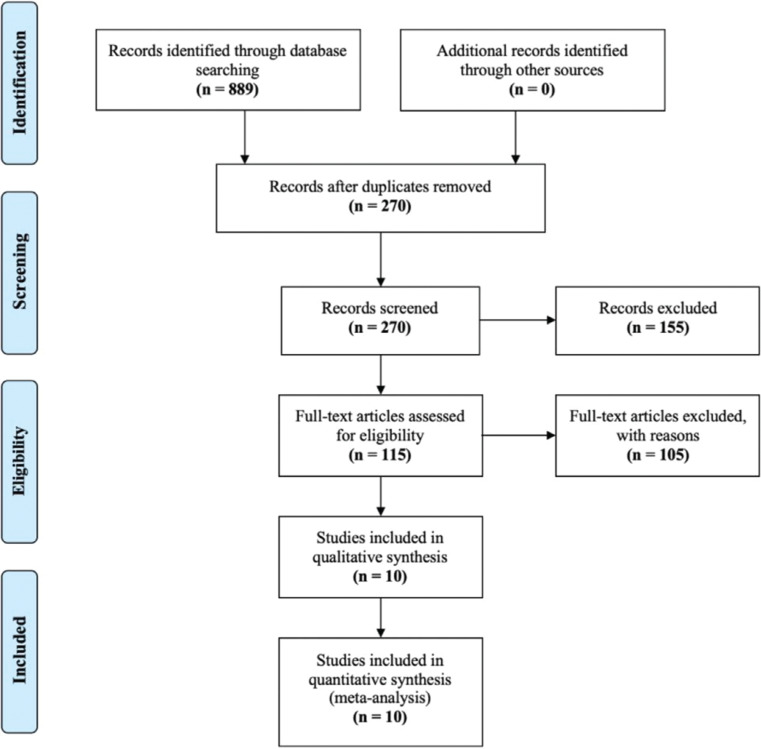
The Preferred Reporting Items for Systematic Reviews and Meta-analysis diagram showing the number of studies screened, number of studies excluded, and number of studies included in this meta-analysis.

A total of 6 RCTs^7,8,16–19^ and 4 observational studies^20–23^ published between 2014–2021 met the inclusion criteria for this meta-analysis **(Figure 1 and Table 1)**. All 6 RCTs were double-blinded, while observational studies included 3 prospective cohort studies and 1 retrospective cohort study. The follow-up period in all studies ranged from 3–36 months, with mean and median follow-up times of 18.2 and 15 months, respectively. A total of 18,548 patients from all the studies were included, of whom 9,328 (50.3%) patients were on ARNIs and 9,220 (49.7%) were on angiotensin-converting enzyme inhibitors (ACEIs)/angiotensin II receptor blockers (ARBs). The mean age of the studied population was 66.7 ± 9.27 years, with the majority of patients (72.7%) being men. All studies except 2 RCTs included HFrEF patients. The PARAGON-HF and Randomized, Double-blind Controlled Study Comparing LCZ696 to Medical Therapy for Comorbidities in HFpEF Patients (PARALLAX) trials involved HFpEF patients. The mean left ventricular ejection fraction among all studied groups was 33.3% ± 8.01%. The majority of patients (60.2%) had ischemic cardiomyopathy. Most patients had New York Heart Association functional class II symptoms (67.3%). In addition to ACEIs/ARBs and ARNIs, most participants reported taking ≥1 additional guideline-directed medical therapy, including β-blockers (87.6% of patients) and mineralocorticoid antagonists (60.7% of patients) **(Table 2)**. Quality and bias assessments of the RCTs and observational studies are included in **Tables 3 and 4**, respectively.

### Outcomes

#### The composite endpoint of sudden cardiac death and ventricular arrhythmias

**Figure 2:**
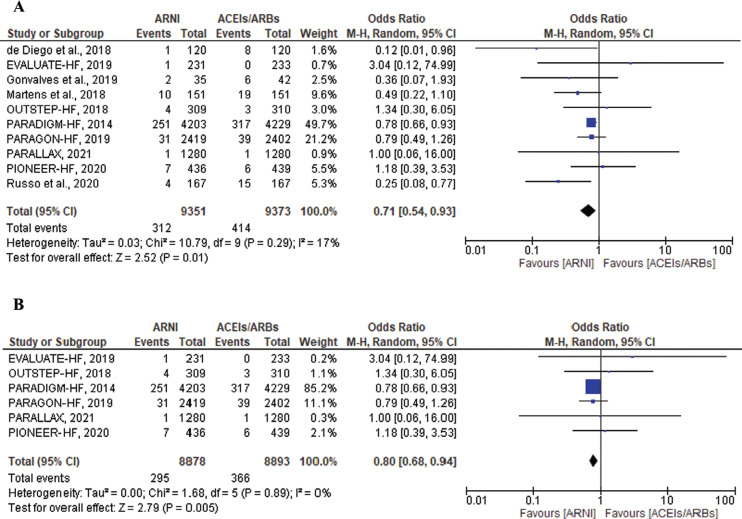
Composite outcome of sudden cardiac death and ventricular arrhythmias among heart failure patients treated with angiotensin receptor–neprilysin inhibitors versus angiotensin-converting enzyme inhibitors/angiotensin II receptor blockers in all included studies **(A)** and in only randomized controlled trials **(B)**. *Abbreviations:* ACEI, angiotensin-converting enzyme inhibitor; ARB, angiotensin II receptor blocker; ARNI, angiotensin receptor–neprilysin inhibitor; CI, confidence interval; M–H, Mantel–Haenszel.

There were a total of 312 events in the ARNI group and 414 events in ACEI/ARB groups of SCD events and ventricular arrhythmias including VT and VF, which was statistically significant (OR, 0.71; 95% CI, 0.54–0.93; *P* = .01; I^2^ = 17%; *P* = .29) **(Figure 2A)**. The PARADIGM-HF trial included a 49.7% weight of the sample size. A sensitivity analysis was performed by excluding the observational studies, resulting in the resolution of heterogeneity. ARNIs showed a significant reduction in the composite of SCD events and ventricular arrhythmias compared to ACEIs/ARBs in the 6 RCTs (OR, 0.80; 95% CI, 0.68–0.94; *P* = .005; I^2^ = 0%; *P* = .89) **(Figure 2B)**.

**Figure 3:**
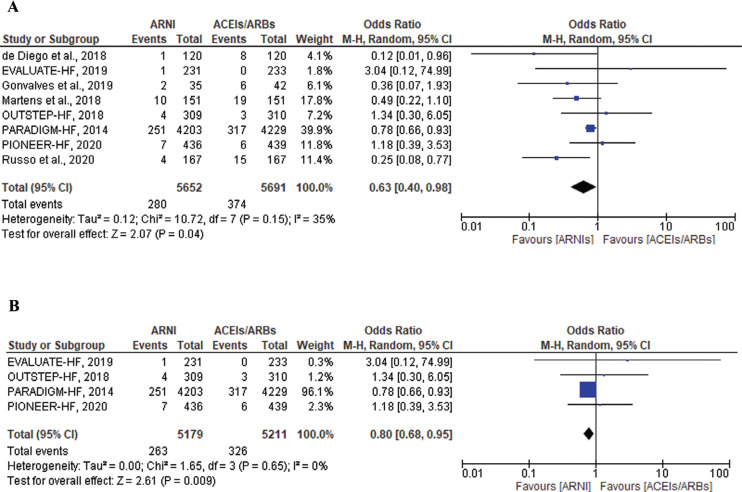
Composite outcome of sudden cardiac death and ventricular arrhythmias among heart failure with reduced ejection fraction patients treated with angiotensin receptor–neprilysin inhibitors versus angiotensin-converting enzyme inhibitors/angiotensin II receptor blockers in all included studies **(A)** and in only randomized controlled trials **(B)**. *Abbreviations:* ACEI, angiotensin-converting enzyme inhibitor; ARB, angiotensin II receptor blocker; ARNI, angiotensin receptor–neprilysin inhibitor; CI, confidence interval; M–H, Mantel–Haenszel.

SCD and ventricular arrhythmias were also analyzed in patients with HFrEF only, which revealed a total of 280 events in the ARNI group compared to 374 events in the ACEI/ARB group (OR, 0.63; 95% CI, 0.40–0.98; *P* = .04; I^2^ = 35%; *P* = .15) **(Figure 3A)**. A sensitivity analysis was performed, excluding the observational studies, which continued to show a significant reduction in the endpoints of SCD and ventricular arrhythmias without any heterogeneity between the groups. A significant reduction in the composite of SCD and ventricular arrhythmia events was observed in the ARNI group compared to the ACEI/ARB group in the 4 RCTs with only HFrEF patients (OR, 0.80; 95% CI, 0.68–0.95; *P* = .009; I^2^ = 0%; *P* = .65) **(Figure 3B)**.

#### Sudden cardiac death

**Figure 4:**
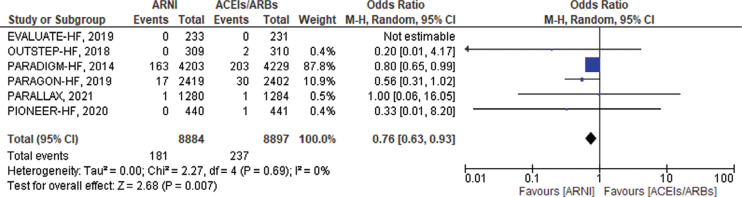
Sudden cardiac death among heart failure patients treated with angiotensin receptor–neprilysin inhibitors versus angiotensin-converting enzyme inhibitors/angiotensin II receptor blockers. *Abbreviations:* ACEI, angiotensin-converting enzyme inhibitor; ARB, angiotensin II receptor blocker; ARNI, angiotensin receptor–neprilysin inhibitor; CI, confidence interval; M–H, Mantel–Haenszel.

SCD outcomes were only available from the 6 RCTs. Most of the events reported were from only 1 trial (PARADIGM-HF with 87.8% weight). A total of 181 SCD, cardiac arrest, or sudden death events were reported in the ARNI group versus 237 events reported in the ACEI/ARB group. SCD was significantly reduced in patients treated with ARNIs compared to ACEIs/ARBs (OR, 0.76; 95% CI, 0.63–0.93; *P* = .007; I^2^ = 0%; *P* = .69) **(Figure 4)**.

#### Ventricular tachycardia

**Figure 5:**
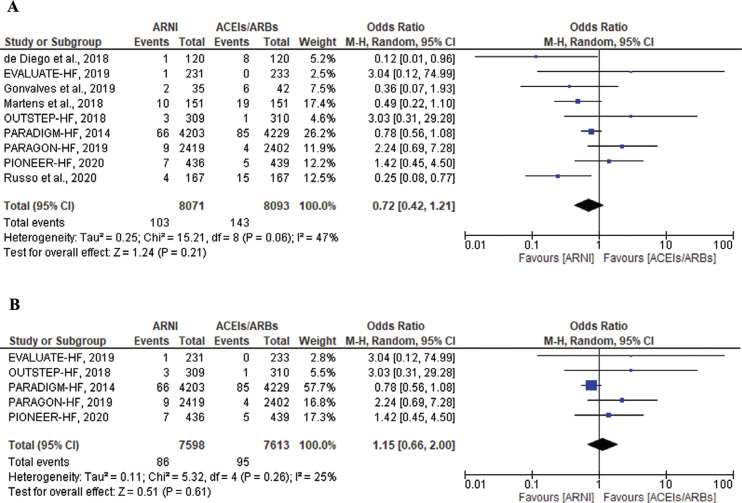
Ventricular tachycardia among heart failure patients treated with angiotensin receptor–neprilysin inhibitors versus angiotensin-converting enzyme inhibitors/angiotensin II receptor blockers in all included studies **(A)** and in only randomized controlled trials **(B)**. *Abbreviations:* ACEI, angiotensin-converting enzyme inhibitor; ARB, angiotensin II receptor blocker; ARNI, angiotensin receptor–neprilysin inhibitor; CI, confidence interval; M–H, Mantel–Haenszel.

There were 103 VT events reported in the ARNI group versus 143 events reported in the ACEI/ARB group. There was no statistically significant difference between the 2 groups (OR, 0.72; 95% CI, 0.42–1.21; *P* = .21; I^2^ = 47%; *P* = .06) **(Figure 5A)**. A sensitivity analysis was performed by excluding the observational studies from the analysis, which showed resolution of the 47% heterogeneity between the groups. However, ARNIs did not lead to a significant reduction in VT compared to ACEIs/ARBs in the 5 RCTs (OR, 1.15; 95% CI, 0.66–2.00; *P* = .61; I^2^ = 25%; *P* = .26) **(Figure 5B)**.

#### Ventricular fibrillation

**Figure 6:**
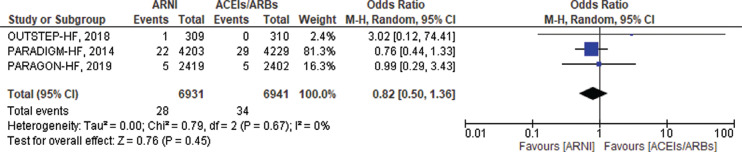
Ventricular fibrillation among heart failure patients treated with angiotensin receptor–neprilysin inhibitors versus angiotensin-converting enzyme inhibitors/angiotensin II receptor blockers. *Abbreviations:* ACEI, angiotensin-converting enzyme inhibitor; ARB, angiotensin II receptor blocker; ARNI, angiotensin receptor–neprilysin inhibitor; CI, confidence interval; M–H, Mantel–Haenszel.

VF outcomes were only available from 3 RCTs. Most of the events reported were from only 1 trial (PARADIGM-HF with 81.3% weight). There were 28 VF events reported in the ARNI group versus 34 events reported in the ACEI/ARB group. There was no significant reduction in the incidence of VF in the ARNI group compared to the ACEI/ARB group (OR, 0.82; 95% CI, 0.50–1.36; *P* = .45; I^2^ = 0%; *P* = .67) **(Figure 6)**.

#### Implantable cardioverter-defibrillator shocks

**Figure 7:**
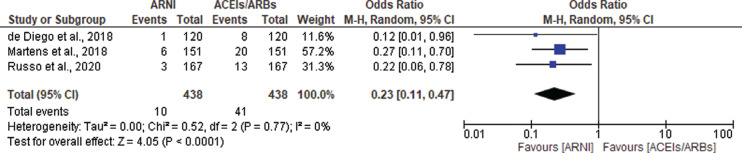
Appropriate implantable cardioverter-defibrillator shocks among heart failure patients treated with angiotensin receptor–neprilysin inhibitors versus angiotensin-converting enzyme inhibitors/angiotensin II receptor blockers. *Abbreviations:* ACEI, angiotensin-converting enzyme inhibitor; ARB, angiotensin II receptor blocker; ARNI, angiotensin receptor–neprilysin inhibitor; CI, confidence interval; M–H, Mantel–Haenszel.

Data on appropriate ICD shocks were available only from the 3 observational studies. There were 10 appropriate ICD shocks in the ARNI group versus 41 in the ACEI/ARB group. The number of ICD shocks was significantly reduced in patients treated with ARNIs compared to ACEIs/ARBs (OR, 0.23; 95% CI, 0.11–0.47; *P* < .0001; I^2^ = 0%; *P* = .77) **(Figure 7)**.

#### Atrial fibrillation/flutter and supraventricular tachycardia

**Figure 8:**
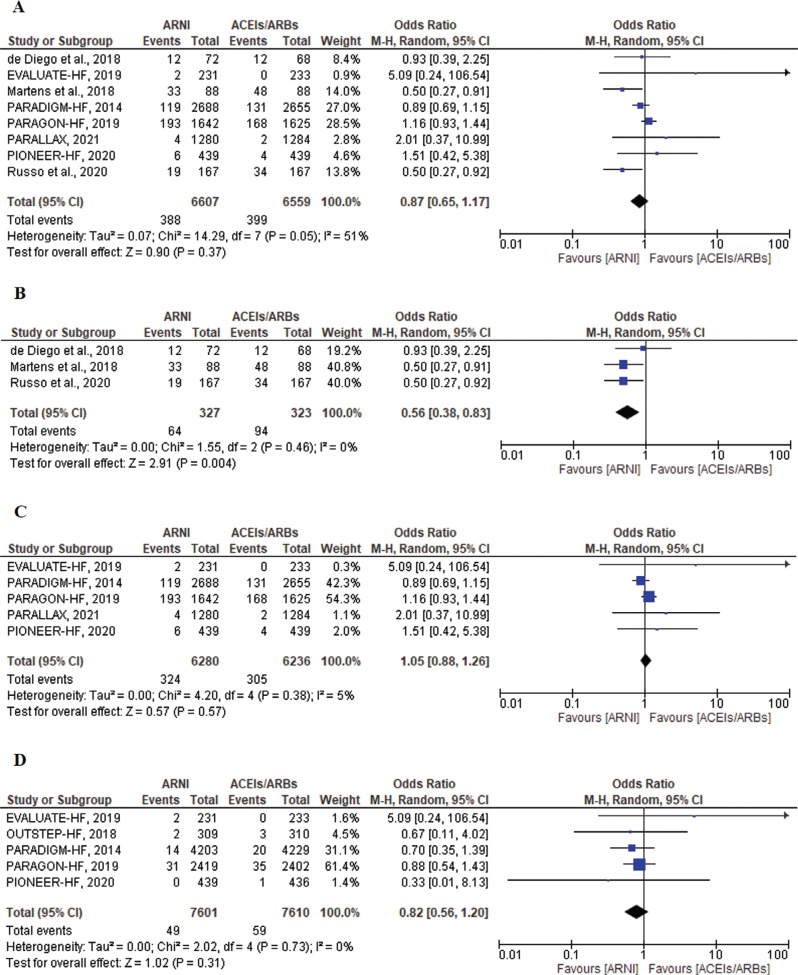
Atrial fibrillation among heart failure patients treated with angiotensin receptor–neprilysin inhibitors (ARNIs) versus angiotensin-converting enzyme inhibitors (ACEIs)/angiotensin II receptor blockers (ARBs) in all included studies **(A)**, observational studies **(B)**, and randomized controlled trials **(C)**. Supraventricular tachycardia among patients treated with ARNIs versus ACEIs/ARBs in randomized controlled trials **(D)**. *Abbreviations:* ACEI, angiotensin-converting enzyme inhibitor; ARB, angiotensin II receptor blocker; ARNI, angiotensin receptor–neprilysin inhibitor; CI, confidence interval; M–H, Mantel–Haenszel.

There was no significant difference in the incidence of AF events between the ARNI group and the ACEI/ARB group (OR, 0.87; 95% CI, 0.65–1.17; *P* = .37; I^2^ = 51%; *P* = .05) **(Figure 8A)**. A subgroup analysis was performed between the RCTs and observational studies due to a moderate heterogeneity of 51%. The observational studies showed a significant reduction in AF in the ARNI group (OR, 0.56; 95% CI, 0.38–0.83; *P* = .004; I^2^ = 0%; *P* = .46) **(Figure 8B)**, which was not evident among the RCTs (OR, 1.05; 95% CI, 0.88–1.26; *P* = .57; I^2^ = 5%; *P* = .38) **(Figure 8C)**. The RCTs also included data on the incidence of SVT. There were a total of 49 events reported in the ARNI group and 59 events in the ACEI/ARB group. There was no significant difference between the groups in terms of SVT events (OR, 0.82; 95% CI, 0.56–1.20; *P* = .31; I^2^ = 0%; *P* = .73) **(Figure 8D)**.

**Figure 9:**
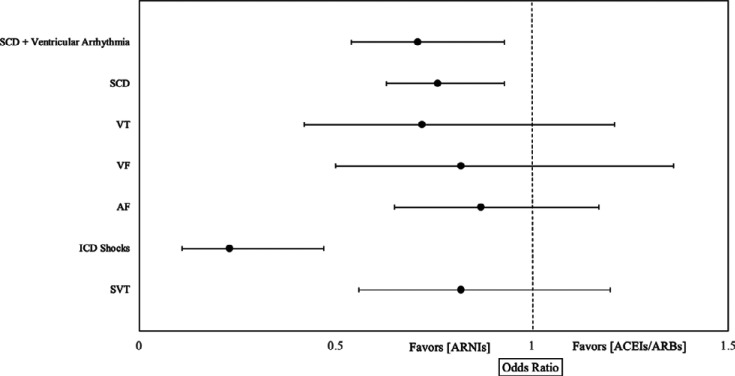
The odds ratios of arrhythmia endpoints for angiotensin receptor–neprilysin inhibitors versus angiotensin-converting enzyme inhibitors/angiotensin II receptor blockers in all included studies for patients with heart failure. *Abbreviations:* ACEI, angiotensin-converting enzyme inhibitor; ARB, angiotensin II receptor blocker; ARNI, angiotensin receptor–neprilysin inhibitor; CI, confidence interval; M–H, Mantel–Haenszel.

Finally, **Figure 9** summarizes the ORs of all arrhythmia endpoints in this meta-analysis for ARNIs and ACEIs/ARBs in all included studies for patients with HF.

## Discussion

This systematic review and meta-analysis demonstrates that patients with HFrEF and HFpEF treated with ARNIs had a lower incidence of the composite endpoint of ventricular arrhythmias (including VT and VF), SCD, and ICD shocks. There were no significant differences in the incidence of AF and SVT between patients treated with ARNIs and those treated with ACEIs/ARBs. The reduction in the composite of ventricular arrhythmias and SCD was mainly driven by the reduction in SCD events as there was no significant difference in isolated VT or VF events. SCD events were only reported in the RCTs, while data regarding ICD therapy were only reported in the observational studies. Significant heterogeneity between the groups was further analyzed using subgroup and sensitivity analyses, and much of the heterogeneity was due to the differences in the type of studies (RCT vs. observational study).

Clinical manifestations of HF are vast, with arrhythmias being one of the most common. Ventricular remodeling resulting in clinical pump failure has been implicated in SCD and strongly correlates with arrhythmia.^24–26^ ARNIs’ theorized effect on arrhythmias involves structural and electrical remodeling on a cardiomyocyte level. In normal hearts, contraction and conformational changes result from membrane depolarization in which calcium (Ca^2+^) enters the cell, resulting in a cascade of events mitigated by the sarcoplasmic reticulum and ryanodine receptor type 2 (RYR2).^25^ Further downstream regulation via sodium (Na^+^)/Ca^2+^ exchanger and Ca^2+^ ATPase pumps results in the sequestration of Ca^2+^, which contributes to diastasis and relaxation.^27,28^ In patients with HF, ventricular myocytes have an upregulated Ca^2+^ removal via the Na^+^/Ca^2+^ exchanger and RYR2 malfunction, which results in calcium leakage, remodeling, and oxidative stress.^29,30^ Ultimately, profound Ca^2+^ imbalance triggers delayed afterdepolarization, resulting in ventricular arrhythmias.^31^ ARNIs have demonstrated the reduction in cardiac remodeling and translational modifications associated with Ca^2+^ homeostasis, which in turn mitigates delayed afterdepolarization, thus reducing ventricular arrhythmias related to SCD.^32,33^

Additional mechanisms of reducing ventricular arrhythmias and SCD via ARNI therapy pertain to the deposition of extracellular matrix proteins and fibrosis associated with electrical inhomogeneity and reentrant ventricular arrhythmias.^34,35^ Studies have demonstrated the reduction in profibrotic markers and myocardial fibrosis in patients after the initiation of ARNI therapy compared to ACEIs/ARBs, in addition to reduction of angiotensin II-mediated cardiac fibrosis and remodeling, which is mitigated by ARNI therapy.^36–38^ The renin–angiotensin–aldosterone system (RAAS), natriuretic peptide, and sympathetic nervous system play essential roles in the progression of HF and ventricular arrhythmogenesis.^39,40^ The inhibition of angiotensin receptors and neprilysin results in the inactivation of RAAS and the natriuretic peptide system, which are overactivated in patients with HF. Neprilysin, a membrane metalloendopeptidase enzyme, is responsible for the degradation of multiple vasoactive peptides and reduces preload and ventricular remodeling.^41,42^ Additionally, ARB blocks the receptor type-1 and decreases the effects of angiotensin II, which prevents vasoconstriction, water retention, and myocardial hypertrophy.^43,44^

To the best of our knowledge, we are the first to conduct a meta-analysis on the outcomes of arrhythmia and SCD in patients with both HFrEF and HFpEF treated with ARNI therapy. This study included an analysis of the composite incidence of ventricular arrhythmias and SCD from RCTs and observational studies.^7,8,16–23^ While composite outcomes of ventricular arrhythmia and SCD were statistically significant, the interpretation of VT and VF separately showed no statistically significant difference. The under-reporting or inaccurate identification of ventricular arrhythmic events may be responsible for the lack of significant statistical outcomes, which is one of the limitations of this study. All studies demonstrated adverse outcomes of tachycardia; however, specification as to whether it was explicitly ventricular was lacking. This inconsistent terminology may be responsible for outcomes favoring a reduction in SCD, but not demonstrating a statistically significant reduction in individual arrhythmias. Additionally, studies did not separate the events in terms of sustained or non-sustained VT, which could be helpful in subgroup analysis. Another limitation of this study includes observational studies with the possible presence of ascertainment bias. Regarding the use of guideline-directed medical therapy, nearly 80% of patients enrolled were on β-blocker therapy, and the use of anti-arrhythmic therapy was not reported in all studies, which could have influenced the results. Thus, further prospective studies are needed to confirm whether such benefits of ARNIs exist.

## Figures and Tables

**Table 1:** Characteristics of Included Studies Enrolling Patients with Heart Failure with Reduced Ejection Fraction or Preserved Ejection Fraction Treated with Angiotensin Receptor–Neprilysin Inhibitors Versus Angiotensin-converting Enzyme Inhibitors/Angiotensin II Receptor Blockers

Study (year)	Study Period (months)	Study Design	Control	Randomization	Intervention/Control	Study Population
PARADIGM-HF (2014)	35	Randomized, double blind	ARB	1:1	4,187/4,212	Age ≥ 18 years, NYHA ≥ II, EF ≤ 35%
OUTSTEP-HF (2018)	3	Randomized, double blind	ACEI	1:1	310/311	Age ≥ 18 years, NYHA ≥ II, EF ≤ 40%
PARAGON-HF (2019)	27	Randomized, double blind	ARB	1:1	2,407/2,389	Age ≥ 50 years, NYHA ≥ II, EF ≥ 45%
PIONEER-HF (2019)	36	Randomized, double blind	ACEI	1:1	440/441	Age ≥ 18 years, EF ≤ 40%
EVALUATE-HF (2019)	3	Randomized, double blind	ACEI	1:1	231/233	Age ≥ 50 years, NYHA I–III, EF ≤ 40%
PARALLAX (2021)	6	Randomized, double blind	ACEI/ARB	1:1	1,280/1,281	Age ≥ 45 years, NYHA ≥ II, EF ≥ 40%
de Diego et al. (2018)^20^	18	Observational, prospective cohort	ACEI/ARB	N/A	120/120	EF ≤ 40%, NYHA ≥ II, ICD
Martens et al. (2019)^21^	12	Retrospective cohort	ACEI/ARB	N/A	151/151	EF ≤ 35%, NYHA ≥ II, ICD or CRT
Gonçalves et al. (2019)^22^	6	Prospective cohort	ACEI	N/A	35/35	NYHA ≥ II, EF ≤ 40%
Russo et al. (2020)^23^	36	Prospective cohort	ACEI/ARB	N/A	167/167	EF ≤ 40%, NYHA II, ICD

*Abbreviations:* ACEI, angiotensin-converting enzyme inhibitor; ARB, angiotensin II receptor blocker; EF, ejection fraction; CRT, cardiac resynchronization therapy; ICD, implantable cardioverter-defibrillator; N/A, not applicable; NYHA, New York Heart Association.

**Table 2:** Baseline Characteristics of the Study Population

NYHA Functional Class, no. (%)				Ischemic Cardiomyopathy, no. (%)	CRT, no. (%)	ICD, no. (%)	Atrial Fibrillation, no. (%)	ACEI or ARB, no. (%)	Mineralocorticoid Antagonist, no. (%)	Digitalis, no. (%)	Diuretics, no. (%)	β-Blocker, no. (%)	Mean EF (%) ± SD	White, no. (%)	Male, no. (%)	Mean Age ± SD (years)	Study
IV	III	II	I														
33 (0.8)	969 (23.1)	2,998 (71.6)	180 (4.3)	2,509 (59.9)	292 (7.0)	623 (14.9)	1,517 (36.2)	3,266 (78)	2,271 (54.2)	1,223 (29.2)	3,363 (80.3)	3,899	29.6 ± 6.1	2,763 (66.0)	3,308 (78.9)	63.8 ± 11.5	PARADIGM-HF
2 (0.65)	146 (47.25)	161 (52.10)	0	177 (57.28)	NA	N/A	147 (47.57)	309 (97.7)	199 (64.4)	0	240 (77.7)	280 (90.6)	NA	298 (96.4)	238 (77.02)	66.9 ± 10.7	OUTSTEP-HF
8 (0.3)	458 (19.0)	1,866 (77.5)	73 (3.0)	899 (37.4)	N//A	N/A	775 (32.2)	2,074 (86.2)	592 (24.6)	N/A	2,294 (95.3)	1,922 (79.9)	57.6± 7.8	1,963 (81.6)	1,239 (48.4)	72.7±8.3	PARAGON-HF
N/A	N/A	N/A	N/A	N/A	N/A	N/A	N/A	208 (47.3)	48 (10.9)	41 (9.3)	292 (66.3)	262 (59.5)	24	262 (59.3)	327 (72.1)	61 ± 14	PIONEER-HF
N/A	N/A	N/A	N/A	N/A	N/A	N/A	N/A	N/A	N/A	N/A	N/A	N/A	N/A	1,112 (86)	1,271 (49.3)	72.6 ± 8.5	PARALLAX
0	100 (21.5)	313 (67.3)	61 (13.1)	283 (60.9)	N/A	N/A	N/A	391 (84)	115 (24.7)	N/A	258 (55.4)	400 (86)	33.5 ± 10	341 (73.5)	355 (76.5)	67.3 ± 9.1	EVALUATE-HF
N/A	N/A	N/A	N/A	98 (82)	52.8 (44)	57.2 (56)	17 (14)	116 (75)	116 (97)	N/A	90 (75)	117 (98)	30.4 ± 4	NA	91 (76)	69 ± 8	de Diego et al.^20^
3 (1.3)	46 (30.7)	102 (68)	0	69	105 (69.6)	46 (30.4)	63 (41)	151 (100)	130 (86)	13 (9)	73 (48)	143 (95)	29 ± 9	NA	123 (82)	67.7 ± 9.9	Martens et al.^21^
N/A	N/A	N/A	N/A	15 (42.9)	7 (20)	30 (85.6)	14 (40)	35 (100)	33 (94.3)	9 (25.7)	N/A	35 (100)	29.3 ± 6.4	NA	29 (82.9)	58.6 ± 11.1	Gonçalves et al.^22^
0	55 (33)	112 (67)	0	86.8 (52.1)	N/A	N/A	34 (20)	167 (100)	150 (90)	N/A	167 (100)	164 (98)	28.1 ± 3.2	NA	140 (84.5)	68.1 ± 11.6	Russo et al.^23^

*Abbreviations:* ACEI, angiotensin-converting enzyme inhibitor; ARB, angiotensin II receptor blocker; CRT, cardiac resynchronization therapy; EF, ejection fraction; ICD, implantable cardioverter-defibrillator; N/A, not applicable; no., number; NYHA, New York Heart Association; SD, standard deviation.

**Table 3:** Quality Assessment of Bias for Included Randomized Controlled Trials

Study	Random Sequence Generation	Allocation Concealment	Blinding of Participants and Personnel	Blinding of Outcome Assessment	Incomplete Outcome Data	Selective Reporting
PARADIGM-HF	Low risk	Low risk	Low risk	Low risk	Low risk	Low risk
OUTSTEP-HF	Low risk	Low risk	Low risk	Low risk	Low risk	Low risk
PARAGON-HF	Low risk	Low risk	Low risk	Low risk	Low risk	Low risk
PIONEER-HF	Low risk	Low risk	Low risk	Low risk	Low risk	Low risk
PARALLAX-	Low risk	Low risk	Low risk	Low risk	Low risk	Low risk
EVALUATE-HF	Low risk	Low risk	Low risk	Low risk	Low risk	Low risk

**Table 4:** Quality Assessment of Bias for Included Observational Studies

Study	Type of Study	Selection	Comparability	Outcome
de Diego et al.^20^	Prospective cohort	★★★★	★★	★★★
Martens et al.^21^	Retrospective cohort	★★★★	N/A	★★★
Russo et al.^23^	Prospective cohort	★★★★	★	★★★
Gonçalves et al.^22^	Prospective cohort	★★★★	★	★★★

*Abbreviation:* N/A, not applicable. Possible maximum of 4 stars for selection, 2 stars for comparability, and 3 stars for outcome, respectively.
